# Supplementary material for: LRRK2 regulates endoplasmic reticulum–mitochondrial tethering through the PERK‐mediated ubiquitination pathway
Source: EMBO J. 2019 Dec 10;39(2):e100875. doi: 10.15252/embj.2018100875 (PMC6960452; doi:10.15252/embj.2018100875)
Supplement: Supplementary file 1 — Appendix [file EMBJ-39-e100875-s001.pdf]

LRRK2 regulates endoplasmic reticulum-–mitochondrial tethering through the PERK-mediated ubiquination ubiquitination pathway

Toshihiko Toyofuku[1,*], Yuki Okamoto[1], Takako Ishikawa[1], Shigemi Sasawatari[1], and Atsushi Kumanogoh[2]
[1] Department of Immunology and Molecular Medicine, Graduate School of Medicine, Osaka University, Suita, Osaka 565-0871, Japan
[2] Department of Respiratory Medicine and Clinical Immunology, Graduate School of Medicine, Osaka University, Suita, Osaka 565-0871, Japan.

Contents

Appendix Figure S1. ER–-mitochondria tethering and Ca^{2+} transients in LRRK2 mutant MEFs

Appendix Figure S2. Generation of genome-engineered MEFs

Appendix Figure S3. Design of pre-miRNA sequence for the BLOCK-iT pol II miR RNAi Expression Vector

Appendix Figure S4. Lists of antibodies used in this study

Appendix Figure S5. Specificity of anti-phospho-serine antibody and Ca^{2+} transients in MEFs transfected with control vectors or treated with vehicle

Appendix Figure S1

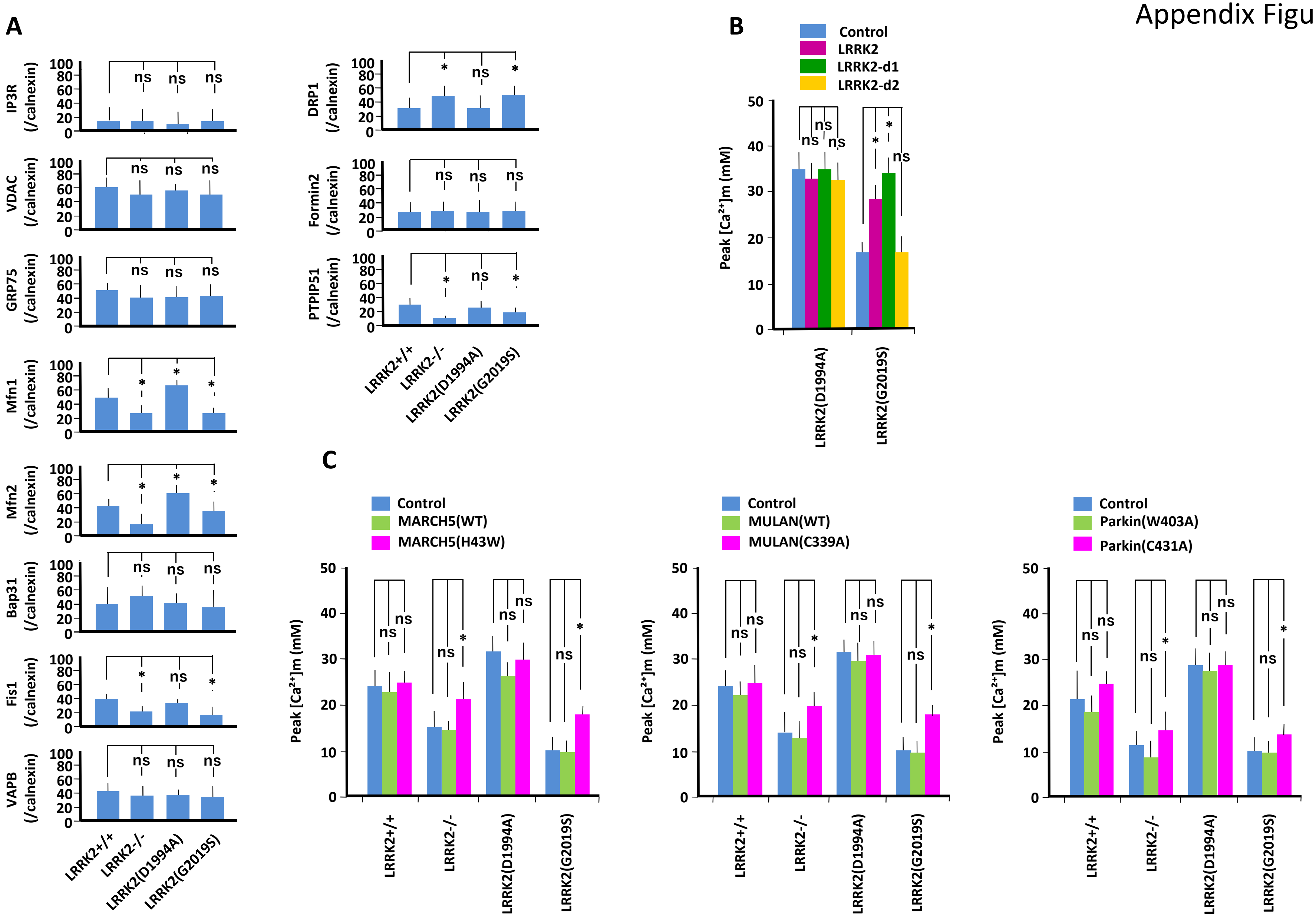

Appendix Figure S1. ER–-mitochondria tethering and Ca^{2+} transients in LRRK2 mutant MEFs

(A) Quantification of mitochondrial tethering proteins, as determined by by immunoblotting. Data represent the ratio of endogenous protein to calnexin levels. Error Error bars represent œ SD from four independent experiments. *P < 0.05 vs. $LRRK2^{+/+}$ MEFs.

(B) Peak values of Ca^{2+} transients in MEFs transfected with LRRK2 deletion constructs. Peak values of Ca^{2+} transients in LRRK2(D1994A) transfected with any of truncated LRRK2 were similar levels, while Peak values of Ca^{2+} transientswhereas the peak value of Ca^{2+} transients in LRRK2(G2019S) transfected with full-length LRRK2 and LRRK2-d1 were was higher than that in LRRK2(G2019S) transfected with empty vector (Control).

(C) Peak values of Ca^{2+} transients in MEFs transfected with shRNAs (shR) [illegible] specific fortargeting E3 ubiquitin ligases. Peak values of Ca^{2+} transients in $LRRK2^{-/-}$ and LRRK2(G2019S) MEFs transfected with shRNAs specific for each of E3 ubiquitin ligases were higher than that that in $LRRK2^{+/+}$ MEFs transfected with empty vector (Control). Error bars represent œ SD from six independent experiments. *P < 0.05 vs. $LRRK2^{+/+}$ MEFs transfected with empty vector (Control).

Appendix Figure S2 ↑

LRRKK2-/-

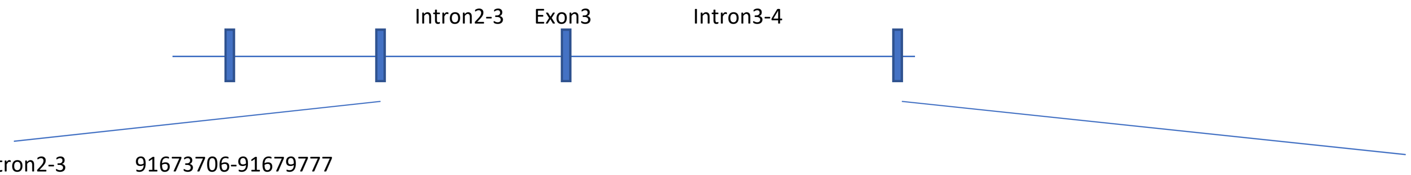

Intron2-3 91673706-91679777
gtaagcgggccttcccatctgttcctgaagactgttctgtgat.......................................(6kb)..taaacaaaaaaggtcaaatctttacctttcttattttcag
Exon3 91679778-91697887
GCGGGGTGGTCACTTCTGTGCAAATTAATAGAAGTCTGTCCAGGGACATTGCAAAGCTTAATAGGACCCCAGGATATTGGAAATGATTGGGAAGTCCTTGGTATTCACCG
Intron3-4 91683092-91697888
Gtaagtgtgatagatacgtgaagataaactgcattgaaaaattatatgt..........................(13kb)...................................ccttcgctttgctgtatgaagttcattgtcatgtgattttgtgatttttattccag

Genomic target CCCAGGATATTGGAAATGAT

Kinase inactive Lrrk2(D1994A) and Lrrk2(G2019S)

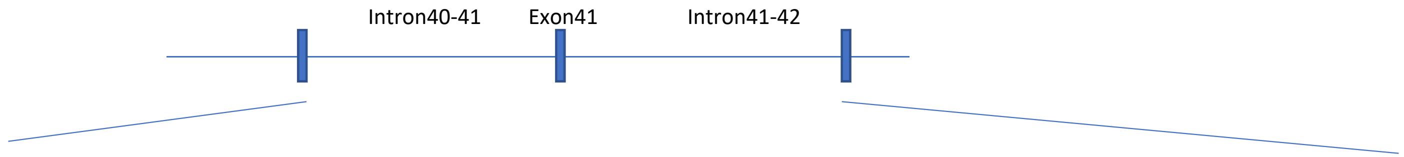

Intron40-41 917800100-91786965
gtaacgggctagcaccggtttctac.......... (7kb)...........tgacgttttgcatgaagaacacaacatattgaaaagaaatctgagcaaattaatataggatgaccgtttttttgataagacctcctgtgtactctctggcag
Exon41 91786966-91787126
GTATCTCCACTCAGCCATGATTATTTACCGTGACCTGAAGCCCCACAATGTGCTGCTTTTTACCCTGTATCCCAATGCTGCCATCATTGCGAAGATTGCGGACTACGGGATCGCACAGTACTGCTGCAGGATGGGAATAAAGACATCAGAGGGCACCCCAG
Intron41-42 91787127-91795996
gtaggagaacaagtttactttttttgcagtggaccatcagtgccctcaagtgtgggtctaggagatatagcctgtca............................(8kb)......................................tattgctgtatctgtttcctttcag

Lrrk2(D1994A)
Genomic target aagacctcctgtgtactctc Mutation GAC to GCG

Lrrk2(G2019S)
Genomic target CGCACAGTACTGCTGCAGGA Mutation GGG to AGC

Appendix Figure S2. Generation of genome-engineered MEFs

(A) Guide RNA for LRRK2 knock-out

LRRK2 genomic target sequence (underlined) was chosen immediately upstream of the protospacer-adjacent motif (PAM, red).

(B) Guide RNA and HDR dsDNA for LRRK2(D1994A)

LRRK2 genomic target sequence (underlined) was chosen due to the close proximity of the cut site to the desired mutation (D1994). HDR dsDNA contains the target mutation (D1994A), a PAM site mutation, and an *Afe*I restriction site to facilitate identification of clones that have undergone HDR.

(C) Guide RNA and HDR dsDNA for LRRK2(G2019S)

LRRK2 genomic target sequence (underlined) was chosen due to the close proximity of the cut site to the desired mutation (G2019). HDR dsDNA contains the target mutation (G2019S), a PAM site mutation, and a *Sfa*NI restriction site to facilitate identification of clones that have undergone HDR.

Appendix Figure S3 ↑

A

IP3R shRNA
IP3R-shR1-top
TGCTG-ACCGAGAGGAGACGCTGTTTA-GTTTTGGCCACTGACTGAC-TAAACAGCGTCTCCTCTCGGT
IP3R-shR1-bottom
CCTG-ACCGAGAGGAGACGCTGTTTA-GTCAGTCAGTGGCCAAAAC-TAAACAGCGTCTCCTCTCGGT-C

IP3R-shR2-top
TGCTG-CTTATGTGAACTTCGTGAATC-GTTTTGGCCACTGACTGAC-GATTCACGAAGTTCACATAAG
IP3R-shR2-bottom
CCTG-CTTATGTGAACTTCGTGAATC-GTCAGTCAGTGGCCAAAAC-GATTCACGAAGTTCACATAAG -C

IP3R-shR3-top
TGCTG-CCCACGGCCATCACCATTAAA-GTTTTGGCCACTGACTGAC-TTTAATGGTGATGGCCGTGGG
IP3R-shR3-bottom
CCTG-CCCACGGCCATCACCATTAAA-GTCAGTCAGTGGCCAAAAC-TTTAATGGTGATGGCCGTGGG-C

PERK shRNA
PERK-shR1-top
TGCTG-CCTCTACTGTTCACTCAGAAA-GTTTTGGCCACTGACTGAC-TTTCTGAGTGAACAGTAGAGG
PERK-shR1-bottom
CCTG-CCTCTACTGTTCACTCAGAAA-GTCAGTCAGTGGCCAAAAC-TTTCTGAGTGAACAGTAGAGG -C

PERK-shR2-top
TGCTG-CCACTTTGAACTTCGGTATA-GTTTTGGCCACTGACTGAC-TATACCGAAGTTCAAAGTGGC
PERK-shR2-bottom
CCTG-CCACTTTGAACTTCGGTATA-GTCAGTCAGTGGCCAAAAC-TATACCGAAGTTCAAAGTGGC-C

PERK shR3-top
TGCTG-CCATGAGTTCATCTGGAACAA-GTTTTGGCCACTGACTGAC-TTGTTCCAGATGAACTCATGG
PERK-shR3-bottom
CCTG-CCATGAGTTCATCTGGAACAA-GTCAGTCAGTGGCCAAAAC-TTGTTCCAGATGAACTCATGG-C

VDAC1 shRNA
VDAC1 shR-top
TGCTG-ACCAGGTATCAAACTGACGTT-GTTTTGGCCACTGACTGAC-AACGTCAGTTTGATACCTGGT
VDAC1 shR1-bottom
CCTG-ACCAGGTATCAAACTGACGTT-GTCAGTCAGTGGCCAAAAC-AACGTCAGTTTGATACCTGGT-C

VDAC2 shR-top
TGCTG-GCTACGGCTTTGGCTTAATAA-GTTTTGGCCACTGACTGAC-TTATTAAGCCAAAGCCGTAGC
VDAC1 shR2-bottom
CCTG-GCTACGGCTTTGGCTTAATAA-GTCAGTCAGTGGCCAAAAC-TTATTAAGCCAAAGCCGTAGC-C

VDAC1-shR3-top
TGCTG-GAGTTGATAAATACCACGTTA-GTTTTGGCCACTGACTGAC-TAACGTGGTATTTATCAACTC
VDAC1 shR3-bottom
CCTG-GAGTTGATAAATACCACGTTA-GTCAGTCAGTGGCCAAAAC-TAACGTGGTATTTATCAACTC-C

B

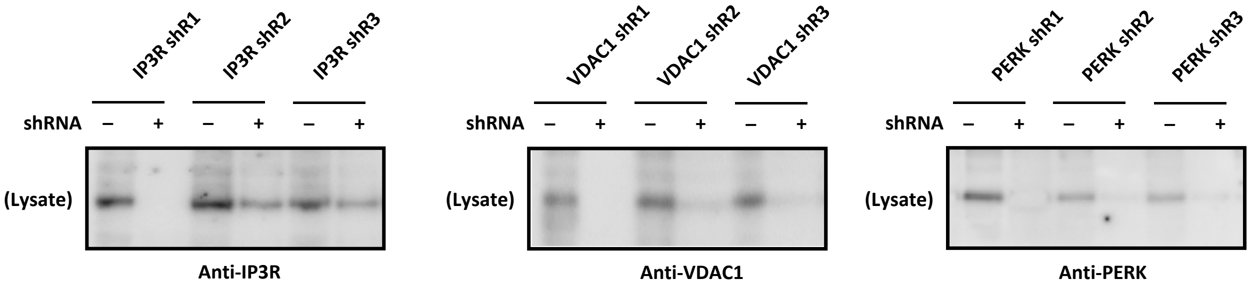

Appendix Figure S3. Design of pre-miRNA sequence for the BLOCK-iT pol II miR RNAi Expression Vector

(A) Nucleotide sequences of shRNAs specific for IP3R, VDAC1, and PERK. shRNA for pcDNA6.2-GW-miR (Clontech Laboratories) was designed by theaccording to the manufacturer's instructions.

(B) Immunoblot of endogenous proteins in transfected MEFs transfected with the indicated shRNAs. Endogenous proteins were effectively decreased knocked down in MEFs by the corresponding shRNAs specific for target protein. Of three different shRNAs, one shRNA, which that suppressed endogenous protein more than 90% , was selected and used in this studyfor use in subsequent experiments..

Appendix Figure S4 ↑

<table>
<tr><td>anti-actin</td><td>Abcam</td><td>ab1801</td><td>https://www.abcam.com/actin-antibody-loading-control-ab1801.html</td></tr>
<tr><td>anti-Bap31</td><td>Abcam</td><td>ab 37120</td><td>https://www.abcam.co.jp/bap31-antibody-ab37120.html</td></tr>
<tr><td>anti-calnexin</td><td>Abcam</td><td>ab22595</td><td>https://www.abcam.co.jp/calnexin-antibody-ab22595.html</td></tr>
<tr><td>anti-DRP1</td><td>Abcam</td><td>ab184247</td><td>https://www.abcam.co.jp/drp1-antibody-epr19274-ab184247.html</td></tr>
<tr><td>anti-Fis1</td><td>Merck Millipore</td><td>MABN391</td><td>http://www.merckmillipore.com/JP/ja/product/Anti-FIS1-Antibody-Clone-4D1.1,MM_NF-MABN391</td></tr>
<tr><td>Anti-INF2</td><td>Proteintec</td><td>11259-1-AP</td><td>https://www.ptglab.com/Products/FMN2-Antibody-11259-1-AP.htm</td></tr>
<tr><td>anti-GRP75</td><td>Abcam</td><td>ab2799</td><td>https://www.abcam.co.jp/grp75--mot-antibody-jg1-ab2799.html</td></tr>
<tr><td>anti-IP3R</td><td>Abcam</td><td>ab125076</td><td>https://www.abcam.co.jp/ip3-receptor-antibody-epr4536-ab125076.html</td></tr>
<tr><td>anti-LC3</td><td>Merck Millipore</td><td>ABC929</td><td>http://www.merckmillipore.com/JP/ja/product/Anti-LC3-I-II-Antibody,MM_NF-ABC929</td></tr>
<tr><td>anti-LRRK2</td><td>Abcam</td><td>ab133475</td><td>https://www.abcam.co.jp/lrrk2-antibody-mjff3-c69-6-ab133475.html</td></tr>
<tr><td>anti-MARCH5</td><td>Abcam</td><td>ab77585</td><td>https://www.abcam.co.jp/march5-antibody-ab77585.html</td></tr>
<tr><td>anti-mitofusin1</td><td>Abcam</td><td>ab57602</td><td>https://www.abcam.co.jp/mitofusin-1-antibody-3c9-ab57602.html</td></tr>
<tr><td>anti-mitofusin2</td><td>Abcam</td><td>ab56889</td><td>https://www.abcam.co.jp/mitofusin-2-antibody-6a8-ab56889.html</td></tr>
<tr><td>anti-MULAN</td><td>ProteinTec</td><td>16133-1-AP</td><td>https://www.biocompare.com/9776-Antibodies/966931-MUL1/?pda=9776|966931_0_0||10|MULAN</td></tr>
<tr><td>anti-Parkin</td><td>Abcam</td><td>ab77924</td><td>https://www.abcam.co.jp/parkin-antibody-prk8-ab77924.html</td></tr>
<tr><td>anti-PTPIP51</td><td>GeneTex</td><td>GTX54674</td><td>https://www.biocompare.com/9776-Antibodies/7588248-PTPIP51-antibody/?pda=9776|7588248_0_0||3|PTPIP51</td></tr>
<tr><td>anti-p62</td><td>abcam</td><td>ab56416</td><td>https://www.abcam.co.jp/sqstm1-p62-antibody-ab56416.html</td></tr>
<tr><td>anti-PERK</td><td>Cell Signaling</td><td>3192</td><td>https://www.cellsignal.com/products/primary-antibodies/perk-c33e10-rabbit-mab/3192</td></tr>
<tr><td>anti-Phospho-PERK</td><td>Cell Signaling</td><td>3179</td><td>https://www.cellsignal.jp/products/primary-antibodies/phospho-perk-thr980-16f8-rabbit-mab/3179</td></tr>
<tr><td>anti-VAPB</td><td>Abcam</td><td>ab103638</td><td>https://www.abcam.co.jp/vapb-antibody-ab103638.html</td></tr>
<tr><td>anti-VDAC1</td><td>Abcam</td><td>ab14734</td><td>https://www.abcam.co.jp/vdac1-porin-antibody-20b12af2-ab14734.html</td></tr>
<tr><td>anti-FLAG</td><td>Sigma-Aldrich</td><td>F1804</td><td>https://www.sigmaaldrich.com/catalog/product/sigma/f1804?lang=ja&region=JP</td></tr>
<tr><td>anti-HA</td><td>MBL</td><td>561</td><td>http://ruo.mbl.co.jp/bio/dtl/A/?pcd=561</td></tr>
<tr><td>anti-Myc</td><td>MBL</td><td>M192-3</td><td>http://ruo.mbl.co.jp/bio/sch/?gtos=8&kw=myc</td></tr>
<tr><td>anti-phosphoserine</td><td>Cell Signaling</td><td>9631</td><td>https://www.cellsignal.com/products/primary-antibodies/phospho-ser-thr-phe-antibody/9631</td></tr>
<tr><td>anti-V5</td><td>Thermo Fisher Scientific</td><td>R960-25</td><td>https://www.thermofisher.com/antibody/product/V5-Tag-Antibody-Monoclonal/R960-25</td></tr>
</table>

Appendix Figure S4. Lists of antibodies used in this study

Appendix Figure S5

A

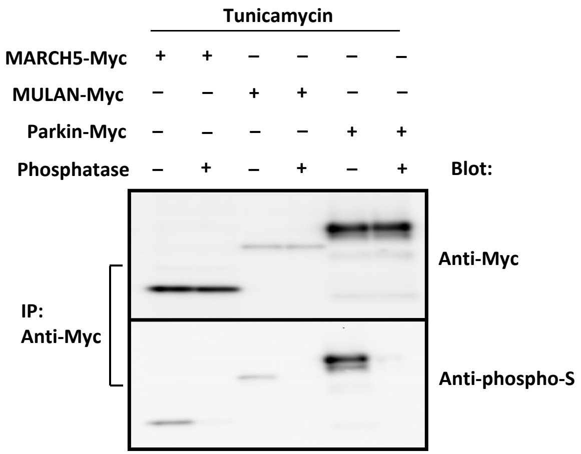

B

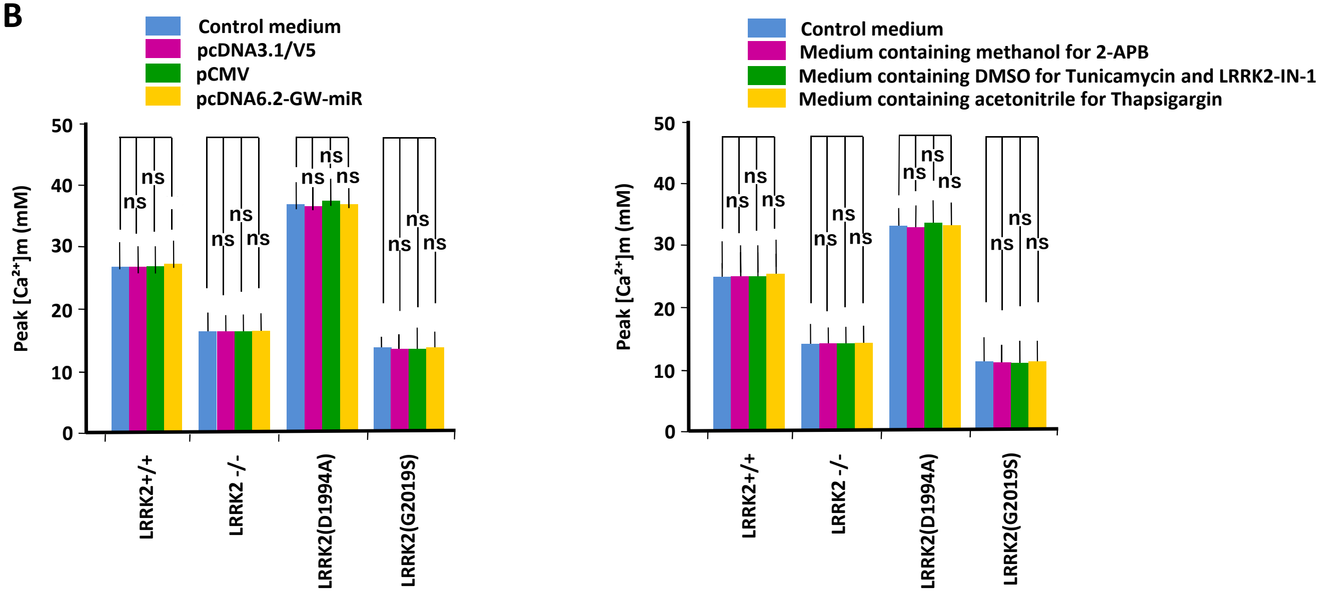

Appendix Figure S5. Specificity of anti-phospho-serine antibody and Ca^{2+} transients in MEFs transfected with control vectors or treated with vehicle

(A) Immunoblot of phosphorylated E3 ubiquitin ligases from transfected MEFs under treated with tunicamycin. Treatment of lysates with phosphatase decreased intensities of bands stained with anti-–phospho-serine antibody, indicating confirming the specificity of this antibody against for phosphorylated proteins.

(B) Peak values of Ca^{2+} transients in MEFs of the indicated genotypes, transfected with expression plasmids (pcDNA3, CMV) or shRNA plasmid (pcDNA6.2-GW-miR) or incubated with the vehicle used for 2-APB, LRRK2-IN-1, and tThapsigargin. All None of the empty vectors and or vehicles had no significant effects on Ca^{2+} transients in MEFs.
