# Supplementary material for: LRRK2 regulates endoplasmic reticulum–mitochondrial tethering through the PERK‐mediated ubiquitination pathway
Source: EMBO J. 2019 Dec 10;39(2):e100875. doi: 10.15252/embj.2018100875 (PMC6960452; doi:10.15252/embj.2018100875)
Supplement: Supplementary file 2 — Expanded View Figures PDF [file EMBJ-39-e100875-s002.pdf]

Expanded View Figures

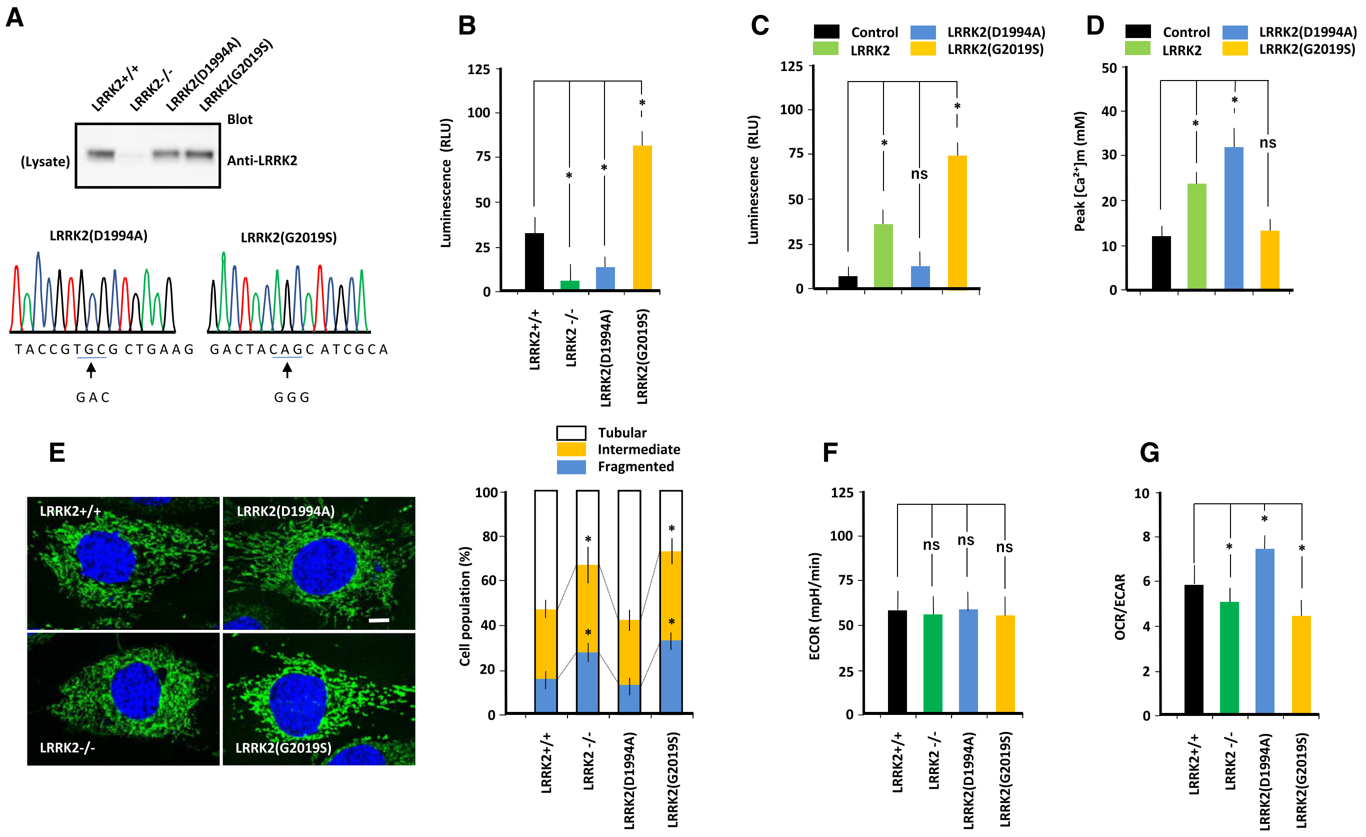

Figure EV1. Functional properties of CRISPR/Cas9-engineered MEFs.

A Properties of LRRK2 in MEFs of the indicated genotypes. (Top panel) Immunoblot of endogenous LRRK2 in MEFs. Results confirm that LRRK2 expression is lost in LRRK2$^{-/-}$ MEFs, and show that LRRK2(D1994A) and LRRK2(G2019S) are expressed at levels similar to wild-type LRRK2. (Middle panel) Nucleotide sequences showing that LRRK2 was mutated at D1994 or G2019 in MEFs.

B Kinase activity of mutant LRRK2 from MEFs, as determined using the ADP-Glo Assay. Data represent kinase activities of LRRK2 prepared from MEFs of the indicated genotypes toward LRRK2-specific substrate (LRRKtide: RLGRDKYKTLRQIRQ). LRRK2(D1994A) had lower kinase activity, whereas LRRK2(G2019S) had higher kinase activity, than wild-type LRRK2. Error bars represent ± SD from eight independent experiments. *$P < 0.05$ vs. LRRK2.

C Kinase activity of mutant LRRK2 from transfected LRRK2$^{-/-}$ MEFs, as determined using the ADP-Glo Assay. Data represent kinase activities of LRRK2 from LRRK2$^{-/-}$ MEFs transfected with mutant LRRK2 toward LRRK2-specific substrate (LRRKtide: RLGRDKYKTLRQIRQ). LRRK2(D1994A) had lower kinase activity than LRRK2(G2019S). Error bars represent ± SD from eight independent experiments.

D Peak values of Ca^{2+} transients in LRRK2$^{-/-}$ MEFs transfected with mutant LRRK2. Peak values of Ca^{2+} transients in LRRK2$^{-/-}$ MEFs transfected with LRRK2(D1994A) were higher than those in LRRK2$^{-/-}$ MEFs transfected with control vector, whereas those in LRRK2$^{-/-}$ MEFs transfected with LRRK2(G2019S) were not significantly different from those in LRRK2$^{-/-}$ MEFs transfected with control vector. Error bars represent ± SD from six independent experiments.

E MEFs of the indicated genotypes were stained with Mitotracker (green) and DAPI (blue). After images were acquired, they were analyzed using a macro developed for the ImageJ software. Mitochondrial morphology was scored as fragmented, intermediate, or tubulated. Data represent the proportions of cells with the indicated mitochondrial morphologies, expressed as percentages of the total number of MEFs counted (≥ 100 cells per experiment). LRRK2$^{-/-}$ and LRRK2(G2019S) MEFs contained more fragmented and less tubulated mitochondria than LRRK2$^{+/+}$ MEFs. Scale bar: 15 μm. Error bars represent ± SD from six independent experiments. *$P < 0.05$ vs. LRRK2$^{+/+}$ MEFs.

F Extracellular acidification rate (ECAR) (mpH/min) of MEFs of the indicated genotypes was measured on an XF24 Analyzer. ECAR values were similar for all MEFs. Error bars represent ± SD from eight independent experiments.

G OCR and ECAR of MEFs of the indicated genotypes. OCR and ECAR were lower in LRRK2$^{-/-}$ and LRRK2(G2019S) MEFs, and higher in LRRK(D1994A) MEFs, than in LRRK2$^{+/+}$ MEFs. Error bars represent ± SD from eight independent experiments.

Data information: For graphs (B–G), the *P* values were determined by a Mann–Whitney *U*-test. ns = not significant, *$P < 0.05$.

Source data are available online for this figure.

 © 2019 The Authors

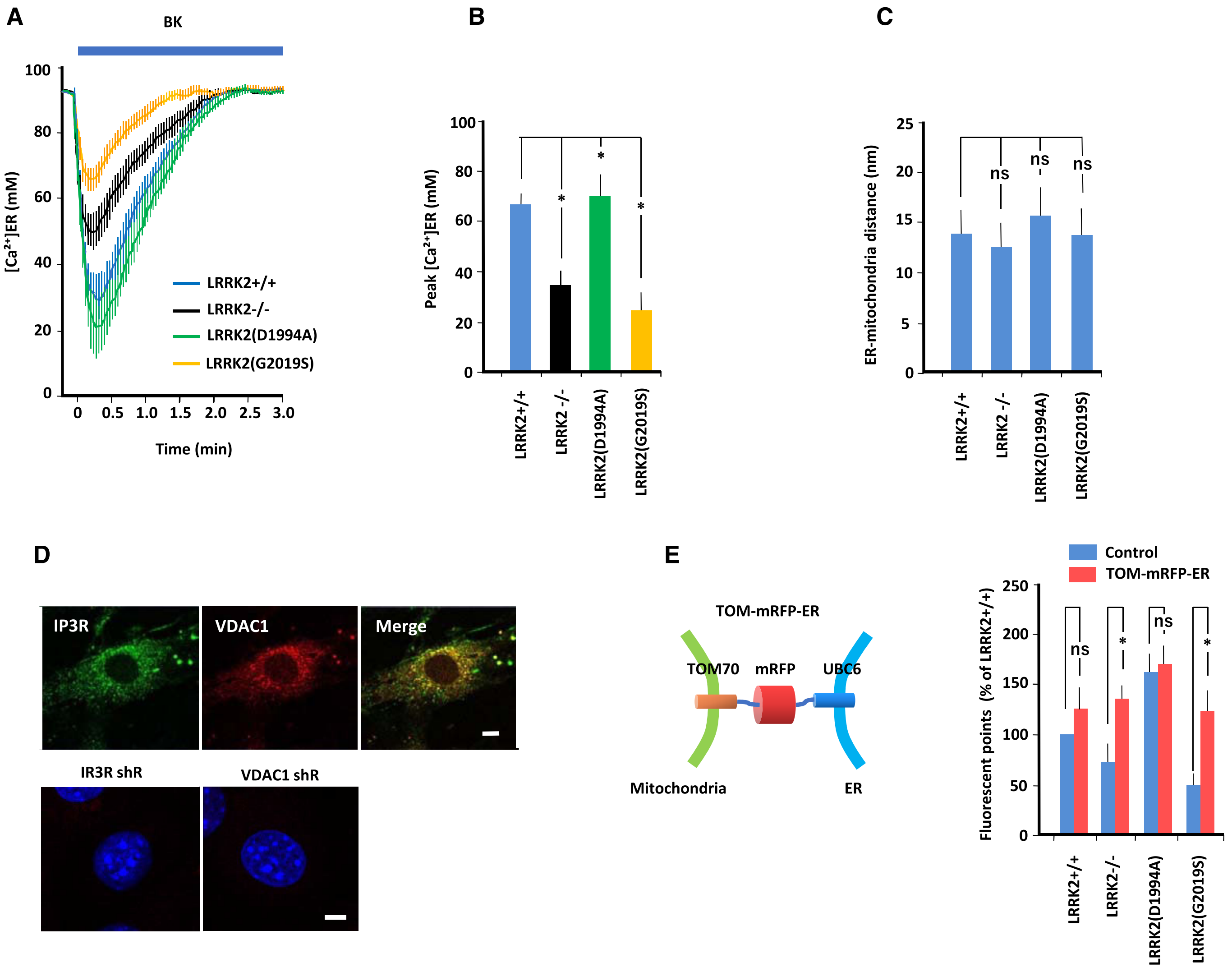

Figure EV2. ER–mitochondrial interaction in MEFs expressing LRRK2 mutants.

A MEFs were transfected with ER-targeted D1ER pcDNA3. Free Ca^{2+} dynamics in the ER matrix ($[Ca^{2+}]_{ER}$) were visualized by FRET. Data are represented as absolute $[Ca^{2+}]$ in μmol. Error bars represent ± SD from eight independent experiments.

B Peak values of $[Ca^{2+}]_{ER}$ in MEFs of the indicated genotype. Peak $[Ca^{2+}]_{ER}$ levels were lower in LRRK2$^{-/-}$ and LRRK2(G2019S) MEFs, and higher in LRRK2(D1994A) MEFs, than in LRRK2$^{+/+}$ MEFs. Error bars represent ± SD from eight independent experiments.

C ER–mitochondrial contacts, as determined by electron microscopy of MEFs of the indicated genotypes. Bar graph shows ER–mitochondrial distance (nm), which was similar in all MEFs. Error bars represent ± SD from 40 images.

D Colocalization of IP3R and VDAC1 in MEFs. (Top panel) Immunofluorescence images of IP3R and VDAC1 in MEFs. MEFs were immunostained with mouse anti-IP3R and rabbit anti-VDAC1 antibodies, followed by anti-mouse IgG-FITC and anti-rabbit IgG-DsRed. Merged image shows the close apposition between ER-localized IP3R and mitochondria-localized VDAC1. Scale bar: 20 μm.
(Bottom panel) *In situ* PLA images of MEFs transfected with IP3R shRNA or VDAC1 shRNA. Loss of either endogenous IP3R or VDAC1 expression dramatically decreased PLA intensity.

E Schematic of synthetic tethering protein (TOM-mRFP-ER) composed of the mitochondrial targeting domain of mouse TOM70, mRFP, and the ER-targeting domain of yeast UBC6. Data represent the results of quantitative analysis of the IP3R–VDAC interaction in MEFs, as determined from *in situ* PLA images using anti-IP3R and anti-VDAC antibodies. PLA intensities in LRRK2$^{-/-}$ and LRRK2(G2019S)-expressing MEFs increased to the intensity in LRRK2$^{+/+}$ MEFs transfected with empty vector (Control). Error bars represent ± SD from six independent experiments.

Data information: For graphs (B, C and E), the *P* values were determined by a Mann–Whitney *U*-test. ns = not significant, $*P < 0.05$.

© 2019 The Authors

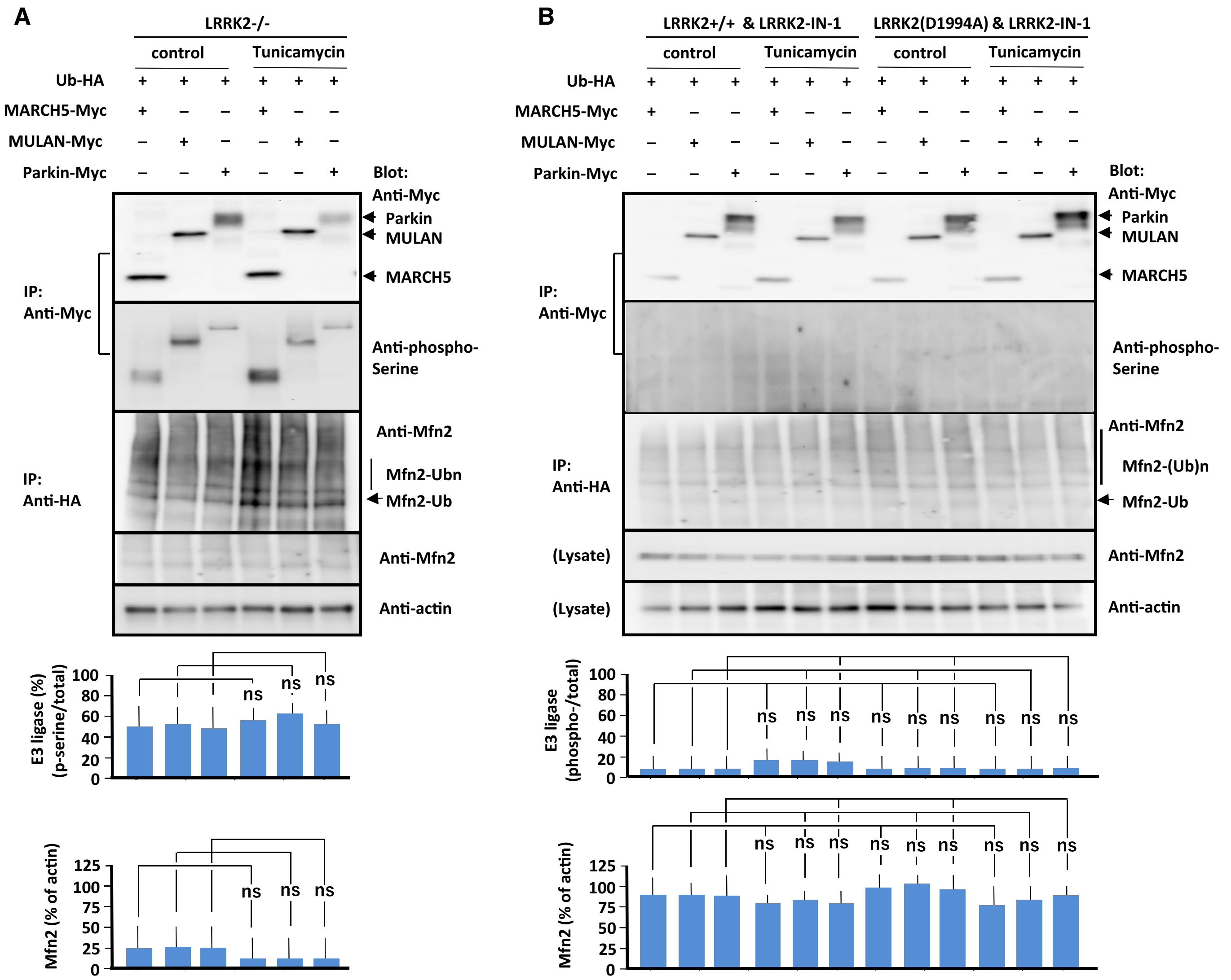

Figure EV3. Effects of loss of LRRK2 or LRRK2-IN-1 on phosphorylation and activation of E3 ubiquitin ligases in LRRK2-mutant MEFs.

A, B Immunoprecipitation/immunoblot of phosphorylated E3 ubiquitin ligases and mitofusin 2 in LRRK2$^{-/-}$ MEFs (A) and MEFs of indicated genotype treated with LRRK2-IN-1 (1 μM) (B) in the absence or presence of tunicamycin (5 μg/ml). MEFs of the indicated genotypes were transfected with each E3 ubiquitin ligase and ubiquitin, and lysates were immunoprecipitated with antibody against the Myc or HA epitope. Precipitated proteins were subjected to SDS/PAGE, and blots were stained with antibody against the Myc epitope, phosphoserine, or mitofusin 2 as indicated to the right of each panel. Data represent the ratios of phosphorylated to total E3 ubiquitin ligase and mitofusin 2 to actin. Treatment of LRRK2-IN-1 blocked phosphorylation of E3 ubiquitin ligases and ubiquitinated mitofusin 2 in LRRK2$^{+/+}$ MEFs in the presence of tunicamycin. Mfn: mitofusin 2, Ub: ubiquitin. Bars represent ± SD from four independent experiments.

Data information: For graphs (A and B), the *P* values were determined by a Mann–Whitney *U*-test. ns = not significant.

 © 2019 The Authors

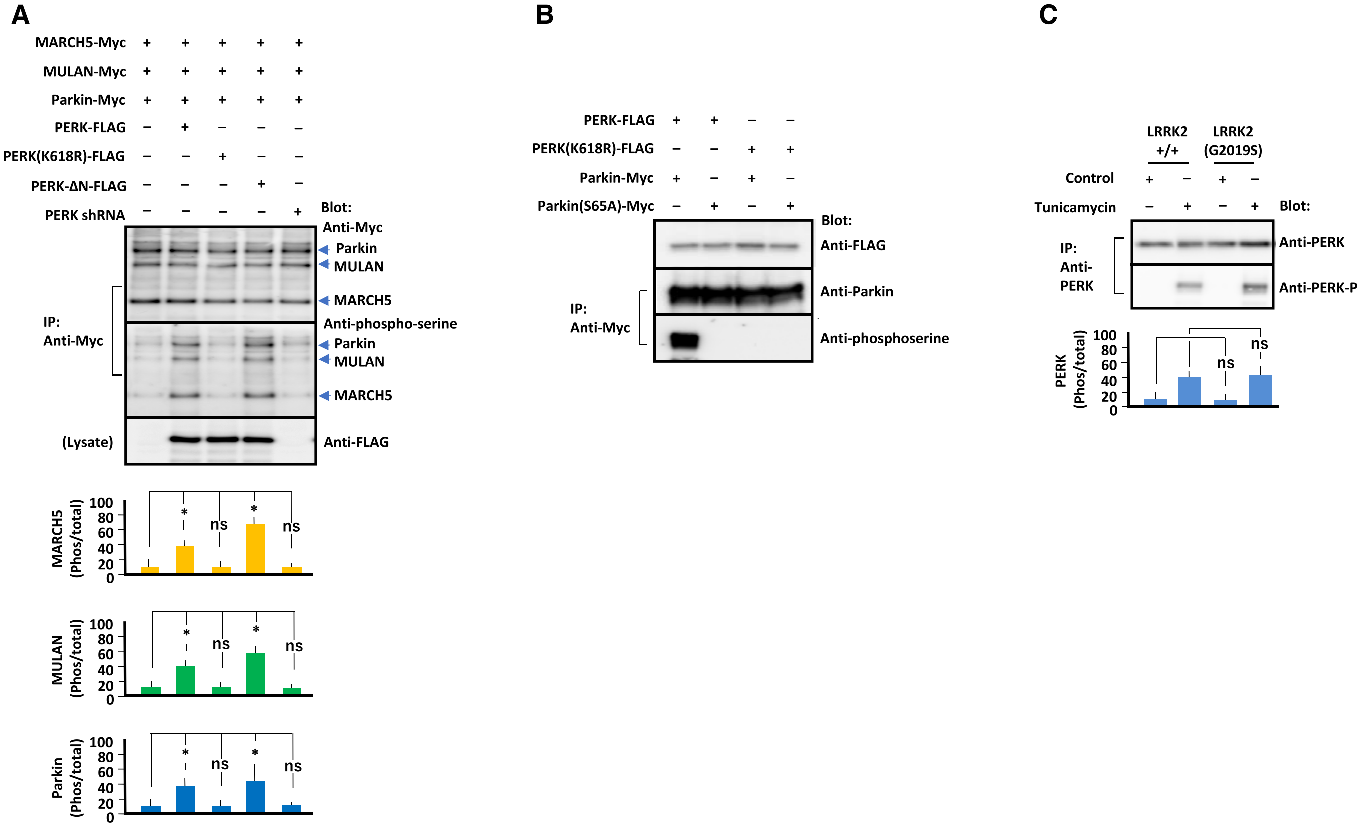

Figure EV4. Phosphorylation of E3 ubiquitin ligases by PERK and phosphorylation of PERK in MEFs under ER stress.

A Immunoprecipitation/immunoblot of E3 ubiquitin ligases phosphorylated by PERK constructs. MEFs of the indicated genotypes were transfected with each E3 ubiquitin ligase, and PERK constructs and lysates were immunoprecipitated with antibody against Myc. Precipitated proteins were subjected to SDS/PAGE, and blots were stained with antibody as indicated to the right. Data represent the ratios of phosphorylated to total E3 ubiquitin ligase to actin. E3 ubiquitin ligases were phosphorylated by PERK or PERK-ΔN, but not by PERK(K618R) or in cells in which PERK had been knocked down by shRNA. Error bars represent ± SD from four independent experiments.

B Immunoprecipitation/immunoblot of Parkin or Parkin(S65A) phosphorylated by PERK or kinase-dead PERK(K618R). HEK293 cells were transfected with Parkin constructs, and PERK constructs and lysates were immunoprecipitated with antibody against Myc. Precipitated proteins were subjected to SDS/PAGE, and blots were stained with antibody as indicated to the right.

C Immunoblot of phosphorylated PERK in LRRK2$^{+/+}$ and LRRK2(G2019S)-expressing MEFs under ER stress. MEFs were treated with vehicle or tunicamycin (1 μg/ml). Endogenous PERK was immunoprecipitated with anti-PERK antibody, and precipitates were immunoblotted with anti-phospho-PERK antibody. Data represent the ratio of phosphorylated to total protein. PERK was phosphorylated in response to tunicamycin treatment at similar levels in LRRK2$^{+/+}$ and LRRK2(G2019S) MEFs. Error bars represent ± SD from four independent experiments.

Data information: For graphs (A and C), the *P* values were determined by a Mann–Whitney *U*-test. ns = not significant, *$P < 0.05$.
Source data are available online for this figure.

© 2019 The Authors

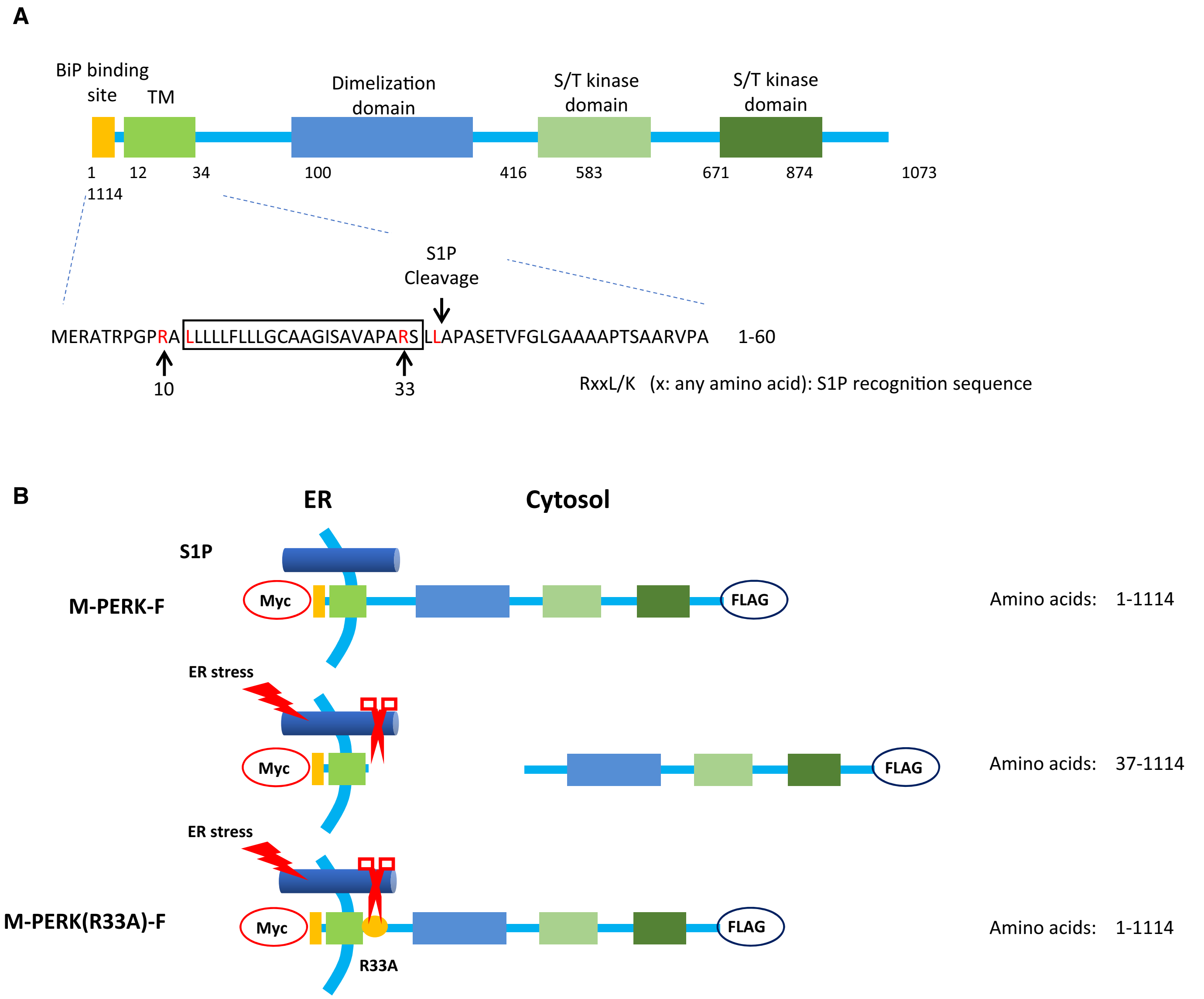

Figure EV5. Amino acid sequence of mouse PERK in the region of the S1P cleavage site.

A Diagram showing full-length PERK. TM, transmembrane domain; S/T kinase, serine/threonine kinase. Amino acid sequences of PERK that are necessary for S1P cleavage are highlighted in red. The box indicates the position of the transmembrane domain.

B Diagram showing full-length PERK tagged with Myc at the N-terminus and FLAG at the C-terminus (M-PERK-F). The S1P recognition sequence R^{33}SLL is mutated to A^{33}SLL (M-PERK(R33A)-F). Under ER stress, Myc-PERK-FLAG is cleaved by S1P at L^{36}-A^{37} and released into the cytosol; the soluble domain is detected by antibody against FLAG, but not Myc.

 © 2019 The Authors
