# Supplementary material for: LRRK2 regulates endoplasmic reticulum–mitochondrial tethering through the PERK‐mediated ubiquitination pathway
Source: EMBO J. 2019 Dec 10;39(2):e100875. doi: 10.15252/embj.2018100875 (PMC6960452; doi:10.15252/embj.2018100875)

Figure EV4A

MARCH5 / MULAN / Parkin

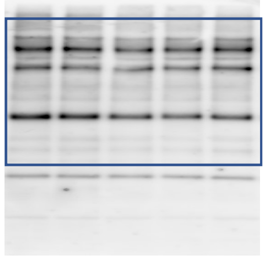

Phosphorylated
MARCH5 / MULAN / Parkin

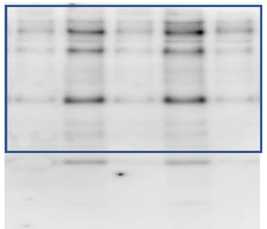

PERK constructs

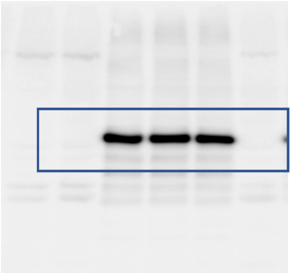

Figure EV4B

PERK constructs

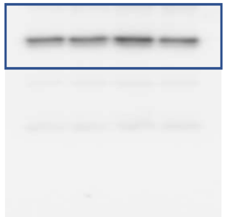

Parkin constructs

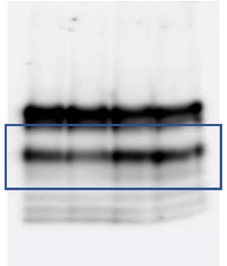

Phosphorylated Parkin

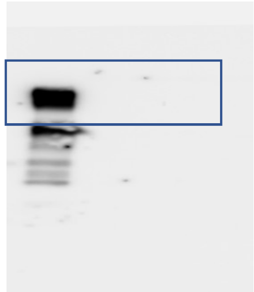

Figure EV4C

Figure EV

PERK

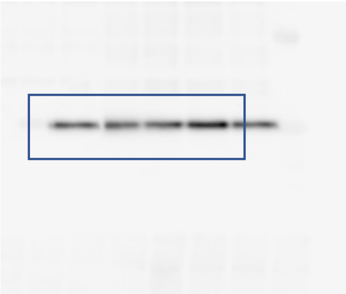

Phosphorylated PERK

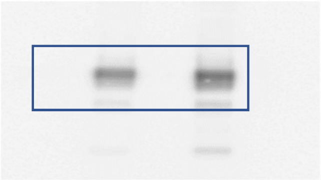

Supplement: Supplementary file 3 — Source Data for Expanded View [file EMBJ-39-e100875-s009.zip › EMBOJ-2018-100875R-SourceDataForFigure_EV4.pdf]
