# Supplementary figures and images for: LRRK2 regulates endoplasmic reticulum–mitochondrial tethering through the PERK‐mediated ubiquitination pathway
Source: EMBO J. 2019 Dec 10;39(2):e100875. doi: 10.15252/embj.2018100875 (PMC6960452; doi:10.15252/embj.2018100875)

## Figure EV1A

LRRK2 mutants

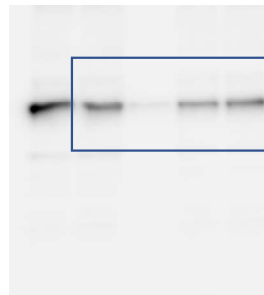

Supplement: Supplementary file 3 — Source Data for Expanded View [file EMBJ-39-e100875-s009.zip › EMBOJ-2018-100875R-SourceDataForFigure_EV1.pdf]

**Figure 1F**

LC3 (Bafilomycin -)

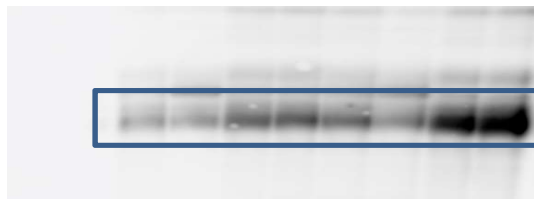

P62 (Bafilomycin -)

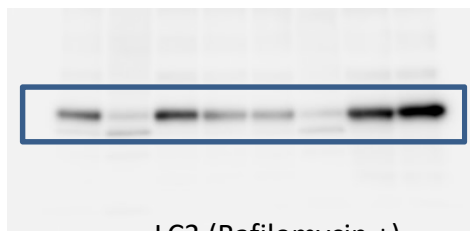

LC3 (Bafilomycin +)

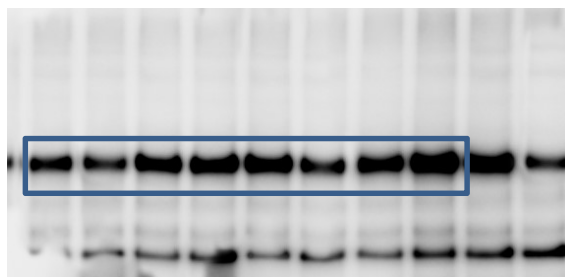

P62 (Bafilomycin +)

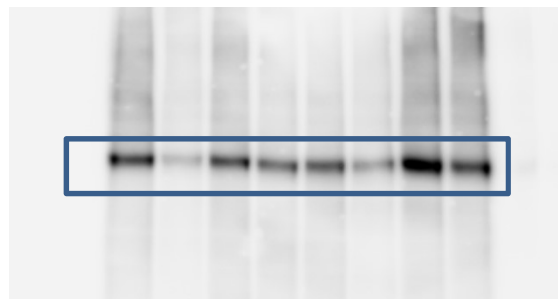

Supplement: Supplementary file 5 — Source Data for Figure 1 [file EMBJ-39-e100875-s003.pdf]

**Figure 3A**

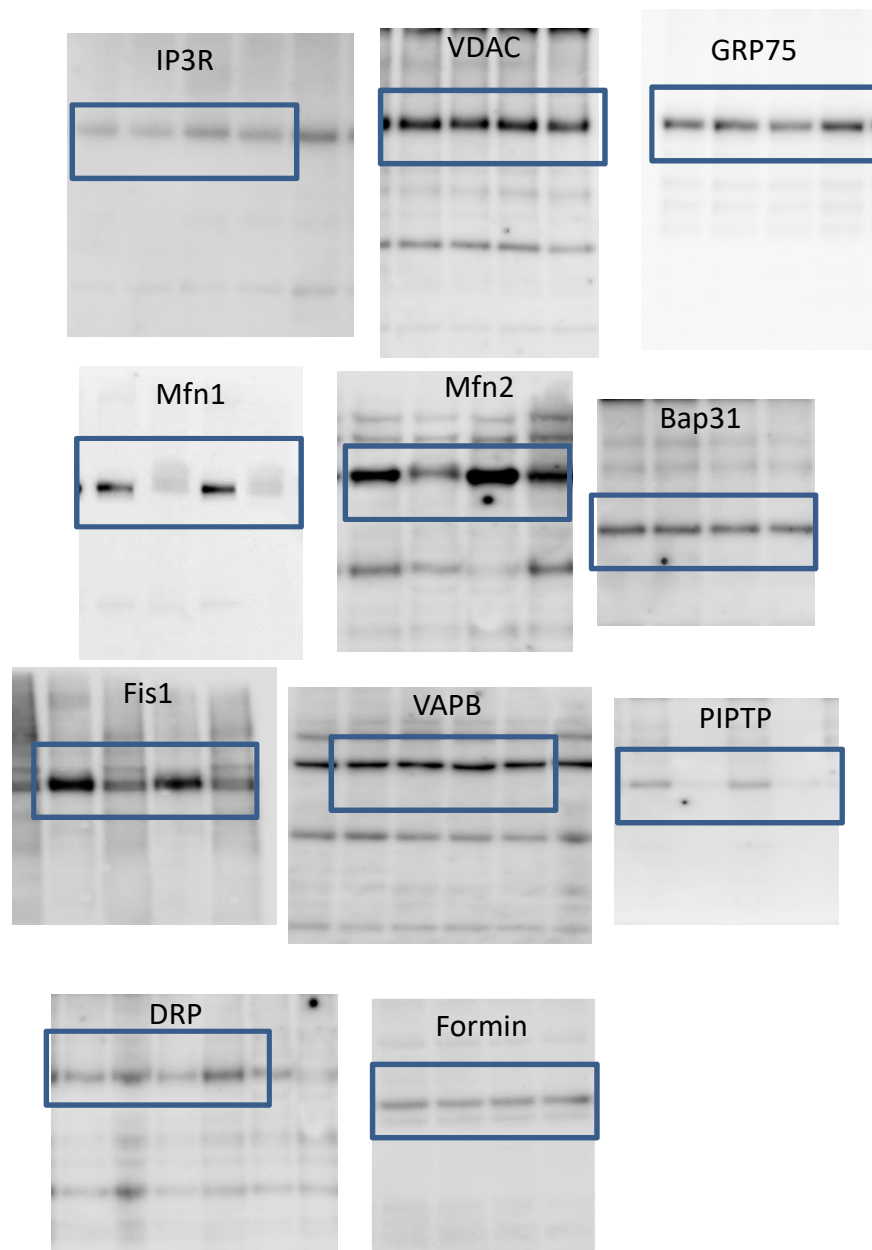

**Figure 3D**

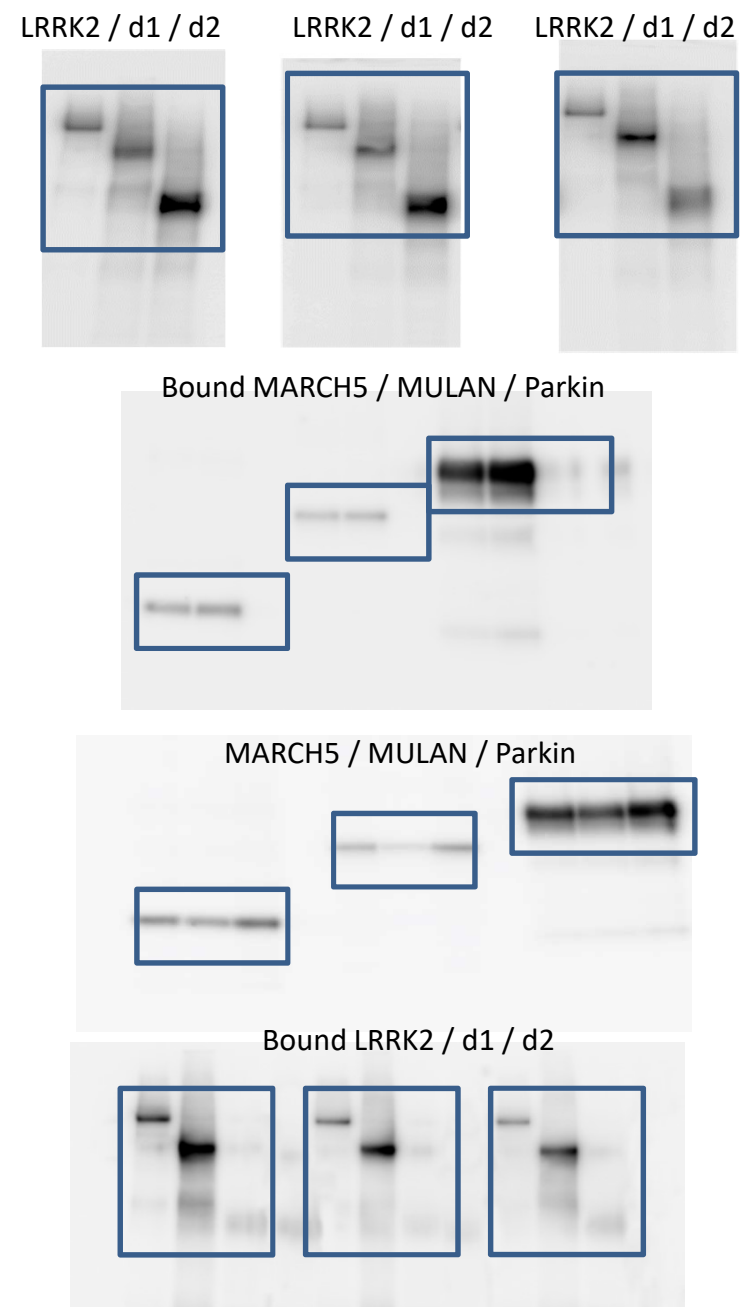

Supplement: Supplementary file 6 — Source Data for Figure 3 [file EMBJ-39-e100875-s004.pdf]

**Figure 4A**

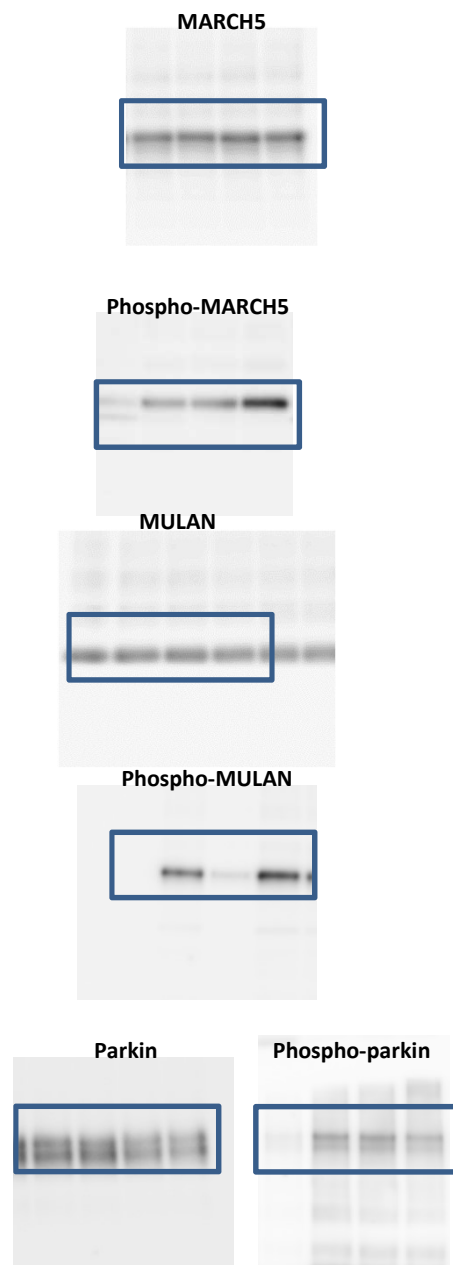

**Figure 4B**

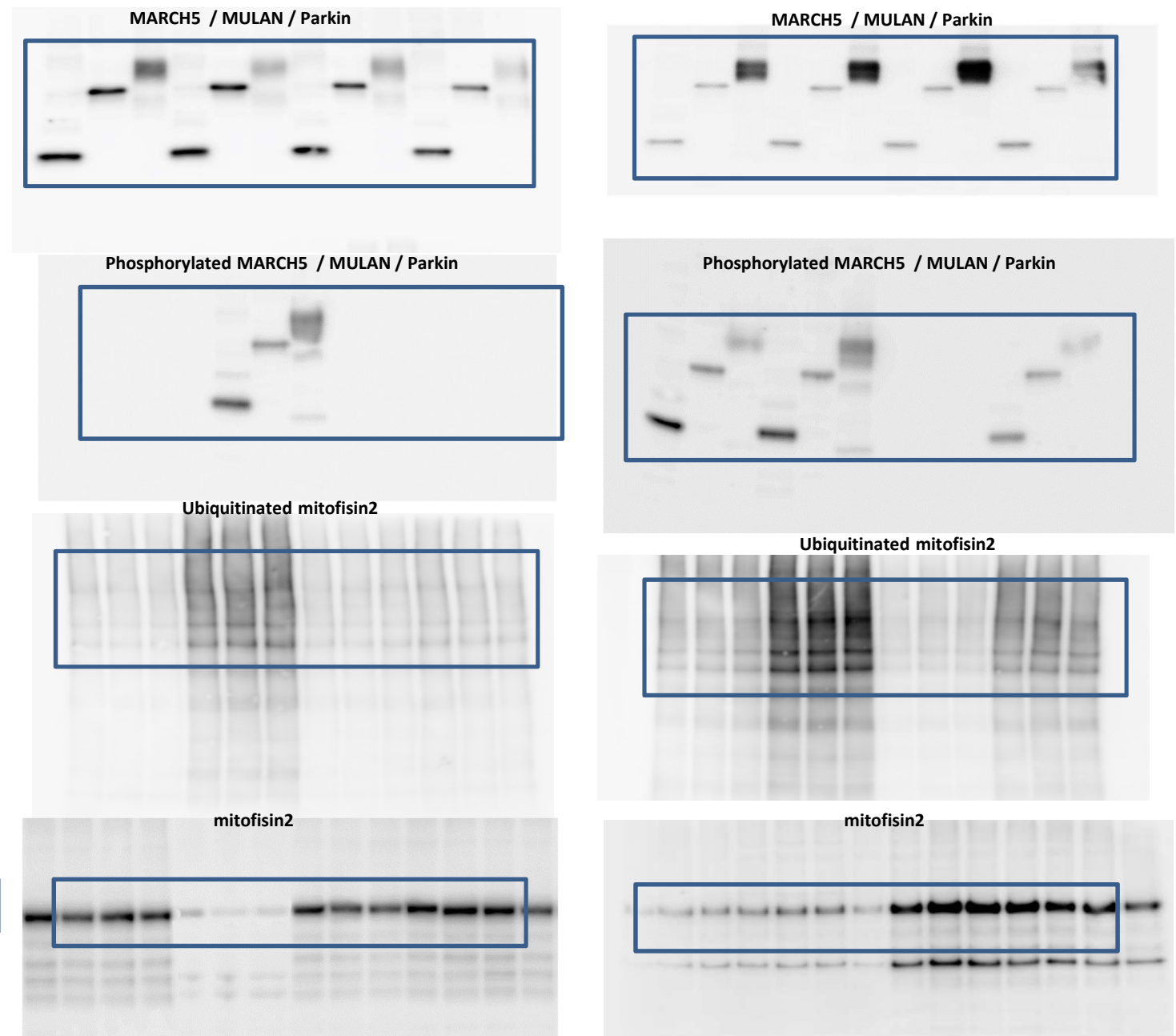

Supplement: Supplementary file 7 — Source Data for Figure 4 [file EMBJ-39-e100875-s005.pdf]

**Figure 5C**

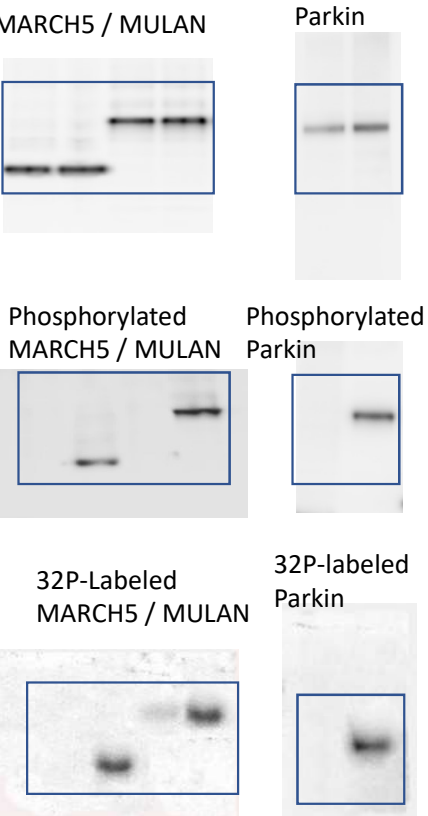

**Figure 5C**

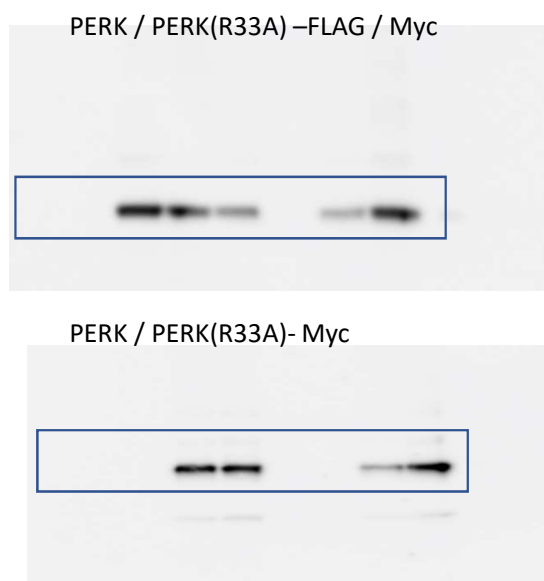

**Figure 5D**

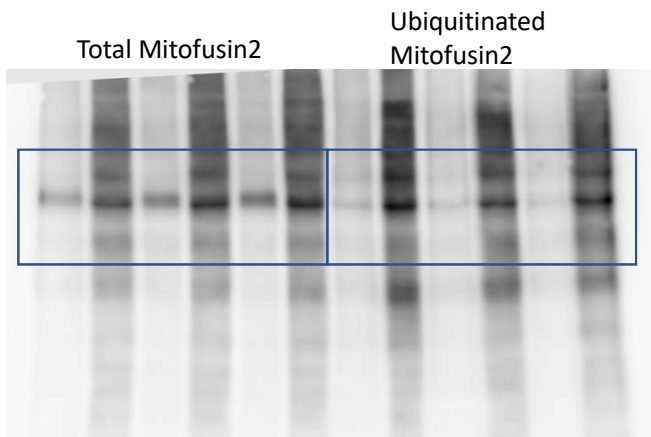

**Figure 5E**

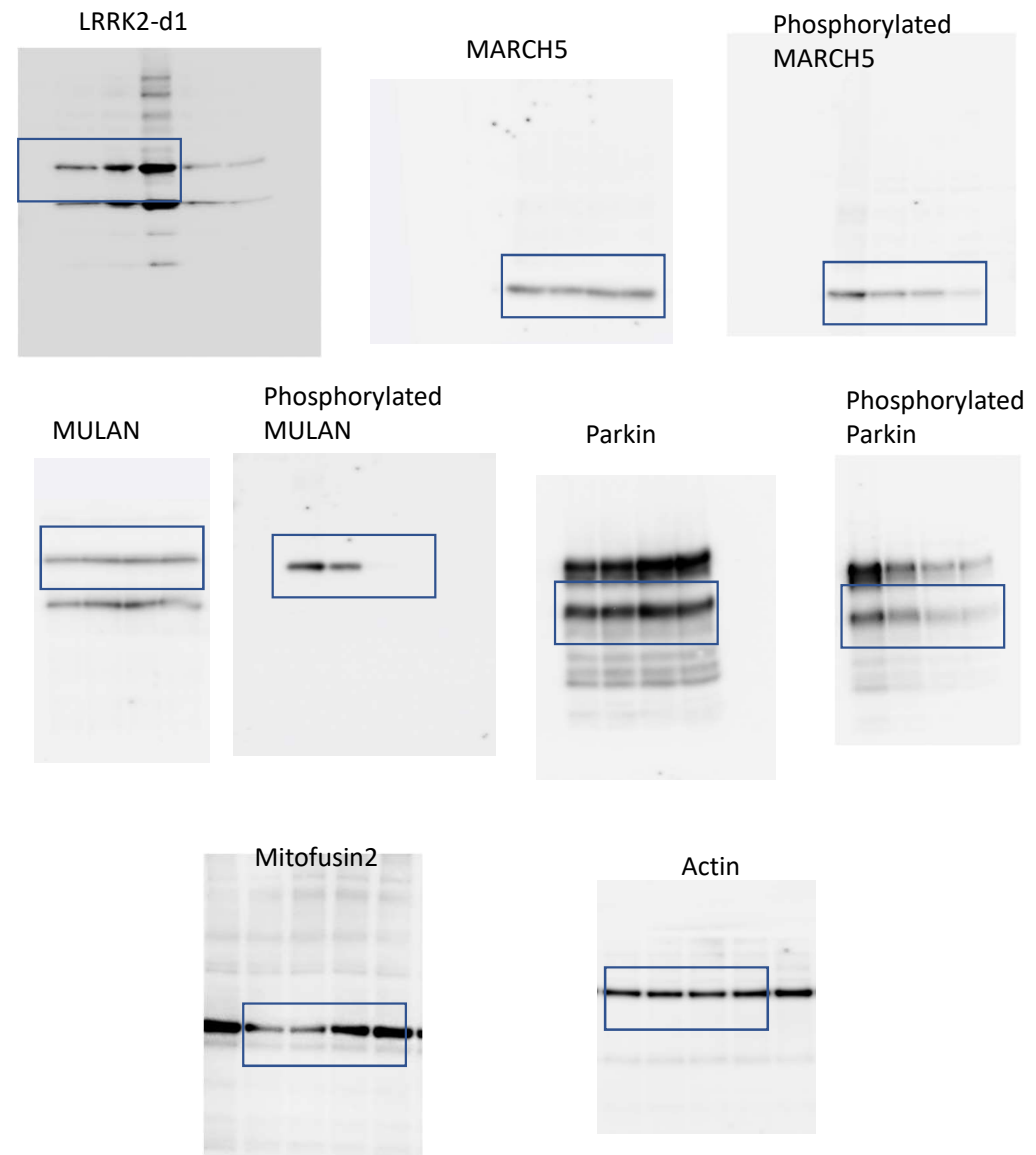

Supplement: Supplementary file 8 — Source Data for Figure 5 [file EMBJ-39-e100875-s006.pdf]

**Figure 7A**

LC3

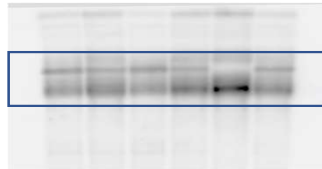

p62

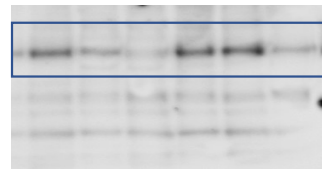

Supplement: Supplementary file 10 — Source Data for Figure 7 [file EMBJ-39-e100875-s008.pdf]
