# Supplementary material for: LRRK2 regulates endoplasmic reticulum–mitochondrial tethering through the PERK‐mediated ubiquitination pathway
Source: EMBO J. 2019 Dec 10;39(2):e100875. doi: 10.15252/embj.2018100875 (PMC6960452; doi:10.15252/embj.2018100875)

Figure 6A

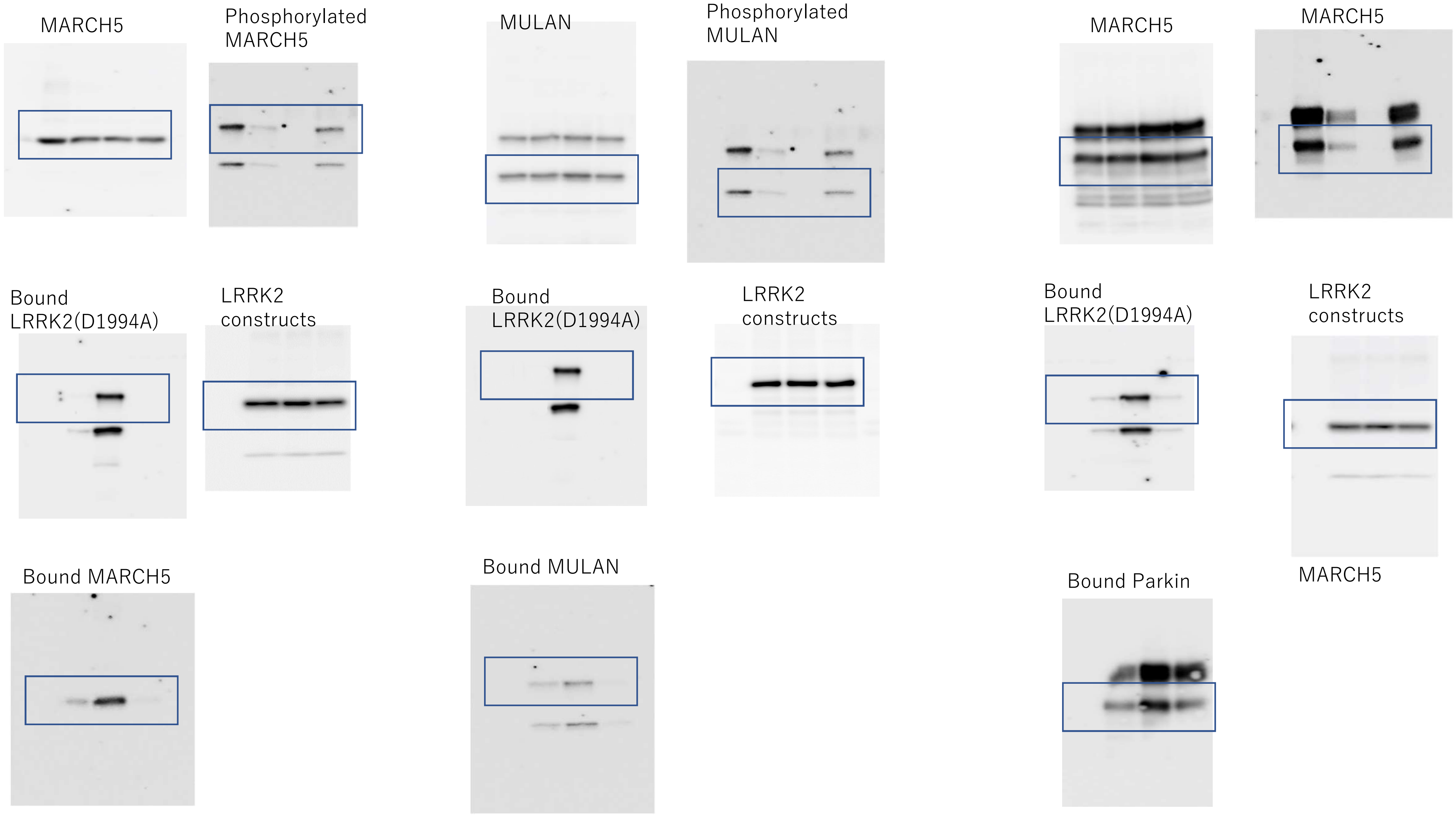
MARCH5
Phosphorylated MARCH5
MULAN
Phosphorylated MULAN
MARCH5
MARCH5
Bound LRRK2(D1994A)
LRRK2 constructs
Bound LRRK2(D1994A)
LRRK2 constructs
Bound LRRK2(D1994A)
LRRK2 constructs
Bound MARCH5
Bound MULAN
Bound Parkin
MARCH5

Figure 6B

LRRK2 constructs

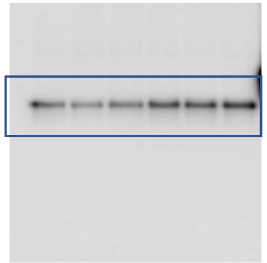

Phosphorylated
LRRK2((G2019S)

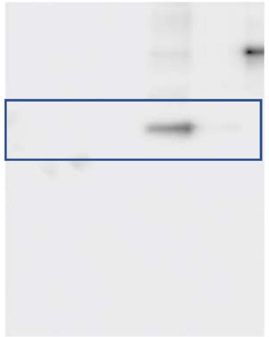

32P-labeled
LRRK2((G2019S)

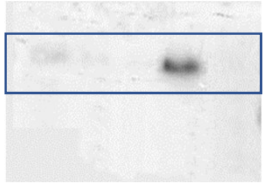

Figure 6C

MARCH5 / MULAN / Parkin

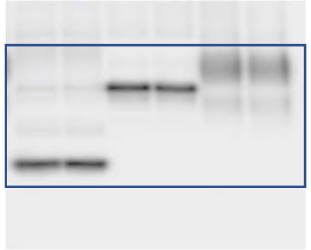

Bound LRRK2(S1290A)

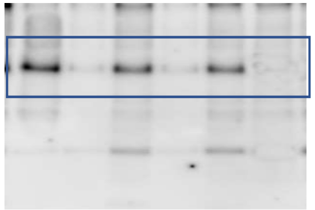

LRRK2 constructs

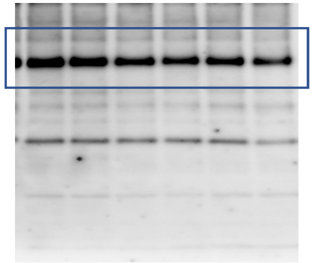

Bound MARCH5 / MULAN / Parkin

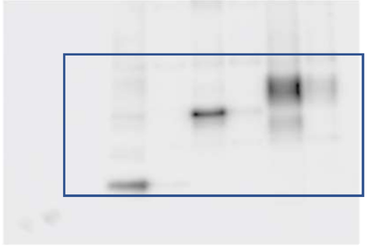

Figure 6D

MARCH5 / MULAN / Parkind2

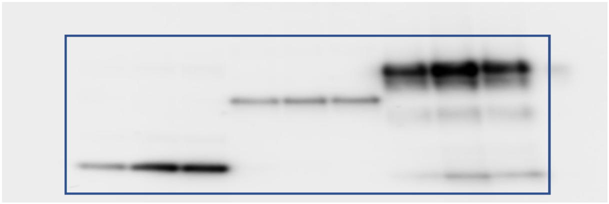

Phosphorylated
MARCH5 / MULAN / Parkind2

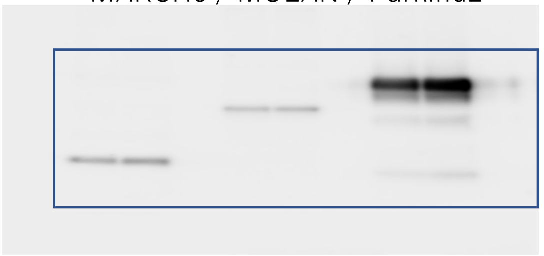

LRRK2 constructs

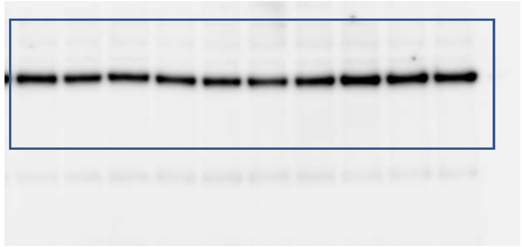

Supplement: Supplementary file 9 — Source Data for Figure 6 [file EMBJ-39-e100875-s007.pdf]
